# Antioxidant Activities of Some Cameroonian Plants Extracts Used in the Treatment of Intestinal and Infectious Diseases

**DOI:** 10.4103/0250-474X.62236

**Published:** 2010

**Authors:** J. Momeni, W. P. Djialeu Ntchatchoua, Fadimatou, M. T. Akam, M. B. Ngassoum

**Affiliations:** Department of Chemistry, Faculty of Science, University of Ngaoundere, P.O. Box 454, Ngaoundere, Cameroon; 1 Department of Applied Chemistry, National Advanced School of Agro-Industrial Sciences (ENSAI), University of Ngaoundere, P.O. Box 455, Ngaoundere, Cameroon; 2 Department of Chemistry, Faculty of Science, University of Buea, P. O. Box 63, Buea, Cameroon

**Keywords:** Antioxidant activity, DPPH and ABTS radical, *Pittosporum mannii*, *Ricinodendron heudelotii*, *Vepris heterophylla*

## Abstract

Antioxidant activity test using two different methods namely 2,2-diphenyl-1-picrylhydrazyl and 2,2'-azinobis(3-ethylbenzothialozinesulfonate) diammonium salt free radical scavenging test has been carried out on three Cameroonian plant extracts used in the treatment of intestinal and infectious diseases: *Pittosporum mannii* Hook f. (Pittosporaceae), *Vepris heterophylla* R. Letouzey (Rutaceae) and *Ricinodendron heudelotii* (Baill) Pierre ex Pax (Euphorbiaceae). Results of this study in the 2,2-diphenyl-1-picrylhydrazyl scavenging test show that the ethyl acetate extract of *P. mannii* and the methanol extract of *V. heterophylla* exhibit high free radical scavenging activities with IC_50_ values of 177.74 and 204.69 μg/ml, respectively while the methanol/dichloromethane (1+1) extract of *R. heudelotii* showed weak free radical scavenging activities as compared to Trolox (939.19 μg/ml) used as standard. In the same manner, 2,2'-azinobis(3-ethylbenzothialozinesulfonate) diammonium salt radical scavenging test of these extracts was in accordance of the result of 2,2-diphenyl-1-picrylhydrazyl test. The antioxidant properties of these extracts probably explain partly, the use of these plants in traditional medicine for the treatment of infectious diseases and inflammations.

In continuation of evaluating *in vitro* biological activities of Cameroonian medicinal plants extracts[1], we report here the antioxidant activities of Cameroonian plants used in the treatment of infectious and intestinal diseases: *Pittosporum mannii* (Pittosporaceae), *Vepris heterophylla* (Rutaceae) and *Ricinodendron heudelotii* (Euphorbiaceae).

*Pittosporum mannii* Hook f. (syn. of *P. viridiflorum* Sims, *P. abyssinicum* Delile var. angolense Oliv., *P. floribundum* Wight and Arn., *P. dalzielii* Hutch. fide Friis) is a shrub widely spread in the savannah zone of Adamawa, in the highlands of West Cameroon and mount Mandara[2]. Known as *Bourangal* in Bororo language, ground internal part of dried bark is given to young child against stomach worm diseases. It is also used in traditional medicine to treat fever, malaria, inflammation and stomach ache and as antidote for insect bites[3–5]. A number of investigations have been performed on *P.* *mannii* indicating that its leaves possess antimicrobial properties and contains compounds such as volatiles monoterpenes, sesquiterpenes, and cytotoxic saponins[3,6,7].

*Vepris heterophylla* R. Letouzey (syn. of *Toddaliopsis heterophylla* A. Engler, *Teclea sudanica* A. Chevalier) is a plant used in folk medicine as diuretic and antipyretic. It is also used for the preservation of foodstuffs in the highlands of Adamawa and the North of Cameroon[8]. Previous studies carried showed that the leaves contains volatile compounds which possess insecticidal properties[9,10], furoquinoline alkaloids[11,12], triterpenes[12] and flavonoids[13].

*R. heudelotii* (Baill.) Pierre ex Pax is a large tree, which grows throughout the humid lowland rainforest of Cameroon[1,14,15]. The bark extract of this plant is used against cough, as poison antidote and for the treatment of intestinal diseases[1,14–16]. The leaves are used to treat dysentery[1,14,15]. The stem bark contains dinorditerpenoids e.g. heudelotenol, heudelotinone as well as E-ferulic acid octacosylate[15] and some natural chemopreventive agents[17].

A survey of literature revealed no report of antioxidant activities on these plants. The aim of this present investigation was to screen the antioxidant properties and to determine if the uses in traditional medicine for treatment of intestinal and infectious diseases can be substantiated through *in vitro* experiments.

Samples of all the above medicinal plants were authentificated at the National Herbarium, Yaounde. Vouchers are kept in the herbarium of laboratories of botany of the Department of Biological sciences of the Faculty of Science of the University of Ngaoundere, Cameroon.

Air dried and ground stem bark of *P. mannii* (1.05 kg) collected from Bafang, West province, Cameroon, was macerated in 5 l of MeOH/CH_2_Cl_2_ (1+1) at room temperature for 72 h. The filtrate was then evaporated to dryness under reduced pressure at 60°. The same operation was repeated three times to give a total extract of 256 g. The residue mixed with 300 ml of water was extracted with ethyl acetate. After concentration as mentioned above, the organic fraction yielded 57 g of ethyl acetate extract which was then extracted with chloroform.

Air-dried and pulverized leaves (500 g) and twigs (150 g) of *V. heterophylla* collected from Tokombere in the far North region of Cameroon near the city of Ngaoundere, Cameroon in October 2006 were extracted at room temperature successively with hexane, ethyl acetate and MeOH. Each time, 100 g of plant material and 3×100 ml of solvent were stirred at 3000 rpm for 4 h. The filtrates were then concentrated to dryness under reduced pressure at 60° to yield respectively hexane (11.43 g; 2.28%), ethyl acetate (16.18 g; 3.23%) and MeOH (16.06 g; 3.21%) extracts from the leaves and hexane (7.18 g; 4.78%), ethyl acetate (10.46 g; 6.96%) and then MeOH (19.29 g; 12.84%) extracts from the twigs.

Leaves of *R. heudelotii* were collected from Buea, South West province, Cameroon in August 2006. Air-dried powdered leaves (1.00 kg) were extracted successively with ethyl acetate and MeOH/CH_2_Cl_2_ (1+1) at room temperature. After removal of the solvent by concentration under reduced pressure at 60°, 57 g of ethyl acetate and 58 g of MeOH/CH_2_Cl_2_ residue were obtained, respectively.

Two milligrams of each extract were dissolved in 100 μl of the respective solvent. Twenty microliters of each solution were spotted on TLC silica gel plates F_254_ and developed in different solvents system. For Alkaloids checking system used were, a polar extracts in hexane-ethyl acetate (60-40) and polar extracts in toluene:ethyl acetate:diethylamine (70:20:10). For phenolic compounds and flavonoids, apolar extracts were checked in cyclohexane:dichloromethane:formic acid:ethylformiate (35:30:5:30) and ethyl acetate:acetic acid:formic acid:water (100:11:11:26) for polar extracts. Plates were sprayed after running with naturstoff reagent/PEG 4000, anisaldehyde/sulphuric acid, alkaloids detection and with a solution of 0.2% 2,2-diphenyl-1-picrylhydrazyl (DPPH) in 96 % ethanol. DPPH, 6-hydroxy-2,5,7,8-tetramethylchroman-2-carboxylic acid (Trolox), potassium persulfate (K_2_S_2_O_8_), 2,2'-azinobis(3-ethylbenzothialozinesulfonate) diammonium salt (ABTS), gallic acid were purchased from Aldrich, Germany, Folin-Ciocalteau phenol reagent, Na_2_CO_3_, methanol (PA) from Sigma Germany. All solvents used for the tests were upgrade. Water used was distilled.

Total phenolic compounds in the ethyl acetate (EA) extract of *P. mannii* and in the MeOH extract of *V. heterophylla* were determined with Folin-Ciocalteu reagent and calculated using gallic acid as the standard. Extracts (100 μl) were added to 50% Folin-Ciocalteau reagent (100 μl). After 3 min, 2 ml of 2% Na_2_CO_3_ solution was added to the mixture, which was then left to stand for 30 min. Absorbance was measured at 750 nm on a spectrophotometer and compared to gallic acid calibration curves. The content of total phenolics is expressed as gallic acid equivalents (GAEs)[18]. All analyses were run in three replicates and averaged.

Five milligrams of dried extracts were dissolved in 10 ml of MeOH to make a stock solution of 0.5 mg/ml. Different concentrations of extracts (500, 250, 200, 100, 50, 25 μg/ml) were prepared by dilution with methanol from this stock solution. DPPH radical scavenging activity of all extracts was determined according to a modified version of De Beer *et al*[19]: 10 μl of each extract, standard Trolox solution (0.025-0.40 mM) or 10% methanol (positive control) was added to 200 μl of a 6.34 10^−5^ M ethanol solution of DPPH; control was prepared without any extract and the absorbance of the reaction mixture at 520 nm was measured after five minutes at 36.5° using a Tecan 5.01 microplates reader. All tests were run in triplicate and averaged. Radical scavenging was expressed as % inhibition of DPPH.

The ABTS scavenging activity of samples were measured according to the method used by Re *et al*[20]. An ABTS^+^ (7 mM) in water was preincubated for at least 12 h with 2.45 mM (final concentration) of K_2_SO_8_ to produce the radical cation. The ABTS^+^ solution was diluted with 96% ethanol to an absorbance of 0.70±0.02 at 734 nm at 30°. Two hundred microliters of ABTS^+^ solution was added to 10 μl of each extract concentration prepared as indicated above, standard Trolox solution (0.025-0.40 mM in EtOH 96%) or 10% methanol used as control. The absorbance of the mixture was determined in the Tecan 5.01 microplatereader after 240 s of incubation at 37°. The absorbance of the resulting oxidized solution was compared to that of the calibrated Trolox standard. Result were expressed in term of TEAC (Trolox equivalent antioxidant capacity) μg/ml. Assays of all samples were conducted in triplicated and averaged.

TLC checks for alkaloids, for phenolics and for terpenoids for all extracts gives the results presented on Table 1. TLC assay of these extracts spraying with 0.2% DPPH in 96% ethanol showed immediately many bleached bands for the EA extract of *P. mannii* and for the MeOH extract of *V. heterophylla* while the MeOH/dichloromethane (1+1) of *R. heudelotii* showed only few bands after hours. The best activity seen in the ethyl acetate extract of *P. mannii* and the result on methanol extract of *V. heterophylla* prompted us to examine the antioxidant activity of these two extracts. Results of the phenolic contents with respect of the percent of gallic acid equivalents (GAEs) showed that both extracts are rich in polyphenolic compounds. The EA extract of *P. mannii* was very rich in phenolic derivatives than the MeOH extract of *V. heterophylla* with 314±4 and 262±6 mg/g GAEs, respectively (Table 2). Based on the DPPH and ABTS radical scavenging assay the above results was confirmed and quantified, showing potent antioxidant properties of the EA extract of *P. mannii* and the MeOH extract of *V. heterophylla*. For both extracts, determination of IC_50_ values in DPPH assay gives (177.39±0.55 μg/ml) and (204.69±5.02 μg/ml) for EA and MeOH extract respectively showing that they are respectively about eight and four time more effective than Trolox (939.19±2.44 μg/ml; higher value but lower antioxidant activity) use as standard compound (Table 3). The EA extract of *P. mannii* and the MeOH extract of *V. heterophylla* strongly scavenged ABTS^.+^ in dose dependent manner with IC_50_ being 331.48±1.94 and 368.20±0.58 μg/ml respectively, showing that both extracts are three time more effective than standard Trolox (Tables 4 and 5). Results compared to Trolox; obviously due to content in phenolics.

**TABLE 1:** QUALITATIVE EVALUATION OF ANTIOXIDANT ACTIVITY OF PLANTS EXTRACTS USING 0.2% DPPH ETHANOLIC SOLUTION AS REVELATOR

Medicinal plants	Parts of plants used	Extracts	Quantities (g)	Result of TLC Spraying with 0.2% DPPH solution
*V. heterophylla*	Leaves	HE	11.43	-
		EA	16.18	+
		MeOH	16.06	++++
	Twigs	HE	7.18	-
		EA	10.46	+
		MeOH	19.29	nd
*P. mannii*	Stem bark	CHCl_3_	17	++
		EA	38	++++
*R. heudelotii*	Leaves	EA	56	-
		MeOH/CH_2_Cl_2_	58	+

++++ strong free radical scavenging properties

++ mild radical scavenging properties

+ weak free radical scavenging properties

- no bleaching of the plate spraying with DPPH solution, n.d. not evaluated, HE n-hexane extract, EA ethyl acetate extract, CHCl_3_ chloroform extract, MeOH methanol extract while MeOH/CH_2_Cl_2_ is the methanol/dichloromethane (1+1) extract

**TABLE 2:** QUALITATIVE COMPOSITION AND TOTAL POLYPHENOL CONTENTS IN DIFFERENTS EXTRACTS

Chemical components	MeOH Extract of *V. heterophylla*	Ethyl acetate Extract of *P. mannii*	MeOH/CH_2_Cl_2_ Extract of *R. heudelotii*
Alkaloids	-	-	-
Steroids and sterols	-	-	+
Terpenoids	-	+	+
Flavonoids	-	+	-
Tannins and phenolic compounds	+	+	+
Total phenolic compounds (mg/g of extract)^a^	262±6	314±4	nd

+ presence

- absence

nd not evaluated

a Values are mean of three experiments ±SD expressed in gallic acid equivalences

**TABLE 3:** ANTIOXIDANT ACTIVITY OF MEOH EXTRACT OF *V. HETEROPHYLLA* AND ETHYL ACETATE EXTRACT OF *P. MANNII* IN DPPH TEST USING TROLOX AS STANDARD

Concentration (μg/ml)	% Inhibition of EA extract of *P. mannii*	% Inhibition of MeOH extract *V. heterophylla*	Concentration of Trolox (μg/ml)	% Inhibition of Trolox
25.00	7.17±0.12	5.76±6.00	62,57	2.50±0.44
50.00	15.51±0.53	12.84±6.20	125,14	4.65±0.21
100.00	29.71±1.02	28.12±5.87	250,28	12.00±0.68
125.00	36.70±0.95	32.61±6.27	500,55	25.18±0.51
250.00	68.82±0.49	58.46±5.02	750,83	41.86±9.83
			1001,10	53.25±2.44
IC_50_ (μg/ml)^a^ Mean±SD	177.39±0.55	204.69±5.02	939.19±2.44	

a Values are mean of three experiments±SD

**TABLE 4:** ANTIOXIDANT ACTIVITY OF ETHYL ACETATE EXTRACT OF *P. MANNII* IN ABTS TEST USING TROLOX AS STANDARD

Concentration (μg/ml)	TEAC of EA extract of *P. mannii*	Concentration Trolox (μg/ml)	Trolox Inhibition
50.00	4.62±2.87	125,14	0.18±0.27
100.00	13.99±1.25	250,28	7.04±1.13
125.00	20.37±8.99	500,55	24.05±0.75
250.00	41.28±1.35	750,83	37.08±0.37
500.00	73.07±0.16	1001,10	49.31±1.55
IC_50_ (μg/ml)^a^ Mean±SD	331.48±1.94	1054.0±0.84	

a Values are mean of three experiments±SD.

**TABLE 5:** ANTIOXIDANT ACTIVITY OF METHANOL EXTRACT OF *V. HETEROPHYLLA* IN ABTS TEST USING TROLOX AS STANDARD

Concentration (μg/ml)	TEAC of MeOH extract of *V. heterophylla* Mean ± SD	Concentration Trolox (μg/ml)	Trolox Inhibition Mean ±SD
200.00	-	125,14	2.01±0.14
250.00	51.79±0.58	250,28	5.97±1.39
300.00	54.53±0.62	500,55	18.92±0.28
400.00	64.04±0.98	750,83	30.54±0.56
500.00	84.22±0.14	1001,10	42.14±0.76
IC_50_ (μg/ml)^a^ Mean ± SD	368.20±0.58	1164.55±0.60	

a Values are mean of three experiments±SD

- not observed

These results are in agreement with those obtained by Gomez in 1983 while examining chemical constituents of Malian leaves of *V. heterophylla*[13]. To the best of our knowledge, this is the first report of the presence of phenolic derivatives in *P. mannii* species and the antioxidant activity in the family Pittosporaceae. These results also showed difference in organic compounds contents of the Cameroonian species of *P. mannii* compared to the same species collected from other African countries such as Madagascar, Kenya and Mali. The genus *Pittosporum* (Pittosporaceae) investigated was shown for the first time to display high antioxidant activity. Its high contains in phenolic derivatives might be responsible for the antioxidant activity and might explain its usage in the Cameroonian folk medicine for the treatment of malaria, intestinal diseases and inflammations.
